# Fiber-Reinforced Composites for Full-Arch Implant-Supported Rehabilitations: An In Vitro Study

**DOI:** 10.3390/jcm13072060

**Published:** 2024-04-02

**Authors:** Luisa De Giorgis, Paolo Pesce, Fabrizio Barberis, Alberto Lagazzo, Francesco Pera, Domenico Baldi, Luigi Canullo, Maria Menini

**Affiliations:** 1 Division of Prosthodontics and Implant Prosthodontics, Department of Surgical Sciences, University of Genova, Largo R. Benzi 10, 16132 Genova, Italy; ludeg94@gmail.com (L.D.G.); paolo.pesce@unige.it (P.P.); domenico.baldi@unige.it (D.B.); maria.menini@unige.it (M.M.); 2 Department of Civil, Chemical and Environmental Engineering, University of Genoa, Via Opera Pia 15, 16145 Genova, Italyalberto.lagazzo@unige.it (A.L.); 3 CIR Dental School, Department of Surgical Sciences, University of Turin, 10126 Torino, Italy; francesco.pera@unito.it

**Keywords:** fiber-reinforced composites, prosthodontics, dental implants, full-arch framework, full-mouth reconstruction

## Abstract

**Background:** Fiber-reinforced composites (FRCs) have been proposed as an alternative to traditional metal alloys for the realization of frameworks in full-arch implant-supported prostheses. The aim of the present in vitro study was to evaluate the deflection under load of seven prostheses endowed with frameworks made of different materials, including different types of fiber-reinforced composites (FRCs). **Methods:** A master cast with four implant analogues in correspondence with the two lateral incisors and the two first molars was used to create full-arch fixed prostheses with the same shape and different materials. Prostheses were made of the following different materials (framework+veneering material): gold alloy+resin (Au+R), titanium+resin (Ti+R), FRC with multidirectional carbon fibers+resin (ICFRC+AR), FRC with unidirectional carbon fibers+composite (UCFRC+C), FRC with glass fibers+resin (GFRC+AR), FRC with glass fibers+composite (GFRC+C), and resin (R, fully acrylic prosthesis). Flexural tests were conducted using a Zwick/Roell Z 0.5 machine, and the deflection of the lower surface of the prosthesis was measured in order to obtain load/deflection graphs. **Results:** Greater rigidity and less deflection were recorded for UCFRC+C and GFRC+C, followed by Ti+R and Au+R. The greatest deformations were observed for resin alone, ICFRC+R, and GFRC+R. The results were slightly different in the incisal region, probably due to the greater amount of veneering material in this area. **Conclusions:** When used to realize full-arch frameworks, Au and Ti allow for predictable mechanical behavior with gradual deformations with increasing load. UCFRC also demonstrated good outcomes and less deflection than ICFRCs when loaded. The GFRC full-arch framework may be a valid alternative, although it showed greater deflections. Further studies are needed in order to evaluate how different prosthesis designs and material thicknesses might affect the outcomes.

## 1. Introduction

Full-arch implant-supported rehabilitation is a widespread and predictable rehabilitation for edentulous patients or patients with hopeless dentition. This treatment modality is often associated with immediate implant loading and has shown optimal clinical outcomes and high patient satisfaction [1,2,3,4,5,6,7,8,9,10,11]. 

The majority of protocols (including the all-on-4 and the Columbus Bridge Protocol) require the insertion of a reduced number of implants (4-6 implants) to be considered sufficient as long as the following two key factors are respected: primary stability and occlusal load control in order to avoid the risk of implant micromotion [4]. 

Overloading might represent a risk factor for implant survival, especially in immediate loading protocols, where implants are loaded before the achievement of osseointegration. However, even once osseointegration is achieved, overloading might increase the risk of peri-implant bone resorption and technical complications [12,13,14].

One of the factors that helps in the control of occlusal loads is the rigid splinting of the implants with a passive-fitting prosthodontic substructure, which prevents micro-movements and optimizes the distribution of loads between the implants, reducing the risk of overload [15]. Hasan et al. conducted a 3D-FEA simulation to compare splinted and non-splinted implants. The results suggested a positive effect on load transmission by splinting the implants, with a reduction of stress levels both on the implant system (up to 64%) and on the peri-implant bone (up to 36%).

With isotropic material, the elastic modulus of the material of which the framework is made can influence the stress levels transmitted to the implant system and peri-implant bone. The materials traditionally most used for implant-supported substructures are metal alloys, which offer sufficient rigidity, even in situations where the prosthetic space is reduced. In fact, the advantages include the ability to make implant-supported full-arch prostheses with more natural aesthetics, avoiding reconstruction of gingival tissues in pink acrylic resin or other materials in the case of low prosthodontic volume.

Titanium and its alloys, as well as zirconia, are also commonly used materials for the fabrication of frameworks with CAD-CAM techniques [16,17,18].

An in vitro study by Ogawa et al. [12] compared implant-supported fixed prostheses made of the following three different materials: acrylic resin, glass fiber-reinforced acrylic resin, and titanium. Under stating loading, maximum bending moments were significantly lower for the titanium framework compared to the fully acrylic and the glass fiber-reinforced prostheses. The higher stiffness of titanium led to a smaller deformation of the prosthesis, thereby resulting in a better distribution of occlusal forces among the supporting implants. This might also reduce the risk of fatigue and possible failures due to overloading of the implant prosthodontic components.

A sufficient stiffness of the prosthesis might be particularly important when a reduced number of implants is used in full-arch rehabilitations, since long-span prostheses should be expected and subjected to flexure.

Indeed, with anisotropic composite materials such as carbon fiber-reinforced composites (CFRCs), the final stiffness of the device is due not only to the fiber modulus but also to the fiber/tow orientation and the geometry of the artifact. This is a direct consequence of adopting anisotropic materials like CFRC instead of isotropic and usually homogeneous materials like metals. The direction of the fibers, as well as the applied patterns, is therefore fundamental to determining the final mechanical properties of the device when dealing with FRC materials.

The 3D finite element analysis by Tribst et al. [19] simulated all-on-4 rehabilitation with the following different framework materials: cobalt chromium, zirconia and polyetheretherketone (PEEK). In contrast with the PEEK framework, zirconia and cobalt chromium concentrated stresses in the framework structure, reducing the stress transmitted to the prosthetic screws and dental implants. 

The production of metal frameworks for fixed implant-supported prostheses involves high costs and long processing times. In addition, the poor adhesive affinity between acrylic resin and metal is often the cause of detachment of the tooth veneer from the underlying frameworks (chipping). This disadvantage is easily solved but represents an inconvenience for the patient.

To reduce costs without sacrificing clinical advantages, the search for alternative materials is always growing.

Zirconia is commonly used, but, besides the high costs, several authors consider its rigidity excessive and a possible cause of increased technical and biological complications in implant-supported rehabilitations, especially in case of full-arch, immediate-loading rehabilitations [20,21,22,23,24].

Fiber-reinforced composites (FRCs) are anisotropic composite materials that could conveniently be bio-applied to provide dynamic strength and fracture resistance, especially in relation to weight [25]. The advantages of FRCs compared to traditional metal alloys and zirconia include reduced costs, ease of repair, the possibility of both analogic and CAD-CAM fabrication techniques, light weight, adhesion to composite resin veneering material, and shock absorption capacity [26]. The main disadvantages in prosthodontic applications are related to the possible interaction of the polymer matrix with body fluids and abrasion in the oral cavity. 

A three-dimensional finite element analysis (3D-FEA) by Menini et al. (Int J Prosthodont 2015) compared loading stresses in an implant-supported full-arch fixed prosthesis without a framework (fully acrylic) with metallic and CFRC frameworks. The highest stresses at the implant level were found in the fully acrylic prosthesis, the lowest stresses were found in the prosthesis with a metallic framework, and intermediate values were found in the prosthesis with a CFRC framework. 

The possibility of optimal adhesion to polymeric veneering materials, such as resin veneering, is considered a further advantage of FRC frameworks. 

In fact, in implant-supported rehabilitations, the shock absorption capacity (and proprioceptive capacity) of the periodontal ligament is missing, and the dampening effect of an elastic veneering material (such as the polymer matrix of a composite material) is considered advantageous by several authors, coupled with the splinting effect of a more rigid framework [21,27].

FRCs consist of fibers embedded in a polymer matrix. The polymer matrix represents the weak phase but allows a firm adhesive bond between the fibers and protection from the possible damaging effects of chemical agents or mechanical trauma, forming a barrier against environmental conditions. It also allows for optimal finishing of the prosthesis surface.

Fibers are the strongest phase and can be continuous or discontinuous. 

The mechanical properties of the final FRC devices can vary greatly depending on the arrangement of the fibers, the type of fiber, the quantity of fibers [28], the type of matrix, the quality of their bonding to the matrix, the fabrication technique used, and the skill of the fabricator; therefore, appropriate and specific training is strongly recommended. 

The tensile strength and elastic modulus of a unidirectionally oriented fiber-reinforced polymer will be lowest when these properties are measured at 90° relative to the longitudinal direction of the fibers, while they will be highest when measured in the longitudinal direction of the fibers [29]. 

A multidirectional arrangement of the fibers allows for the distribution of properties over multiple dimensions. Most likely, when the “woven” configuration is adopted, mechanical properties are decreased compared to the longitudinal properties of unidirectionally oriented composites; in any case, it must be considered that it works in the 2D configuration. 

A study by Pesce et al. [30] investigated the mechanical properties of carbon fiber-reinforced composite frameworks by comparing unidirectional and multidirectional fibers. Following destructive and nondestructive mechanical tests to evaluate the static and dynamic elastic modulus, it was revealed that composites with unidirectional carbon fibers are suitable for the fabrication of frameworks for full-arch implant-supported rehabilitations, with a higher dynamic elastic modulus than that of composites with multidirectional carbon fibers, which instead presented a higher static elastic modulus. This is a consequence of the specific features of the dynamic vs. static tests [30].

Most FRCs used in dental applications are fabricated with glass fibers for aesthetic reasons (in contrast with black carbon fibers) and for their surface chemistry, which improves their adhesion to the polymer matrix [31]. The application of glass fiber-reinforced composites ranges from the fabrication of endodontic posts to implant prosthodontics using both analogic and CAD/CAM techniques [32]. Great attention has to be paid to the fact that CFRCs are anisotropic in their mechanical/thermal response; while the most commonly adopted GFRCs in dental applications are constituted of short fibers randomly dispersed into the supporting polymer matrix, and this dramatically changes the overall attended mechanical performance.

Nakamura et al. [33] examined the flexural strength and elastic modulus of three glass fiber-reinforced composites for framework fabrication compared to three veneer composites. The values recorded for framework composites were three times higher than those of veneer composites.

FRC bridges with glass fibers can become an alternative to restorations with metal frameworks. Some studies have demonstrated high fracture toughness with reliable marginal fit after thermal cycling and mechanical loading [34].

However, the application of FRCs in the implant prosthodontic field has not yet been sufficiently investigated in the literature, requiring further studies.

The purpose of the present in vitro study was to compare the mechanical characteristics of seven implant-supported full-arch prostheses made with frameworks and veneers of different materials through flexural tests. In particular, the flexion of the different prostheses was measured when a vertical load was applied.

## 2. Materials and Methods

The samples of this study consist of 12-unit screw-retained upper full-arch fixed prostheses supported by 4 implants at the two lateral incisors and the two first molars. 

**Figure 1:**
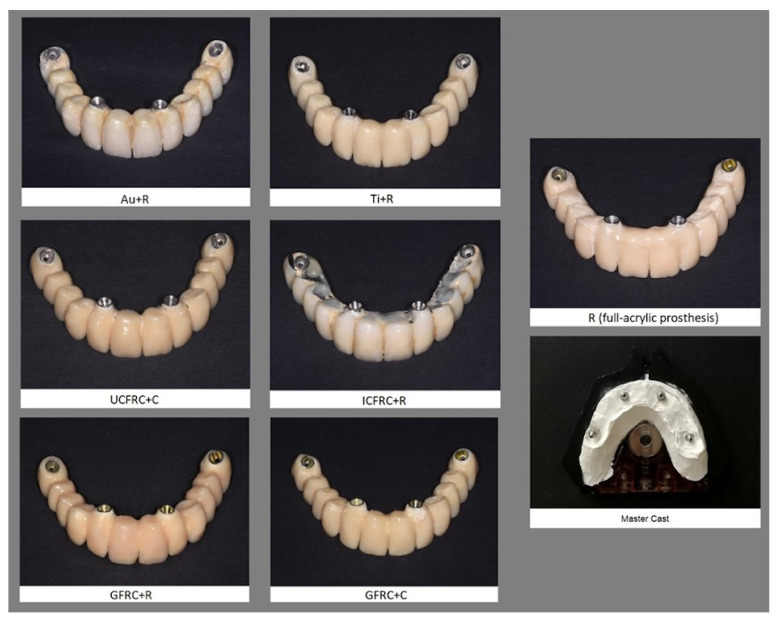
The seven samples examined in this study.

All the prostheses have the same shape and size and were fabricated from one extra-hard plaster model of the upper maxilla (Figure 1) that included the analogs of 4 angled abutments (Biomet 3i, PalmBeach Gardens, FL, USA) with a 4 mm diameter, a 17° inclination at the level of the lateral incisors, and a 30° inclination at the level of the first molars to form an optimal polygonal support. The distance between the implants at the lateral incisor sites was 19.4 mm. The distance between the lateral incisor and the first premolar was 27.6 mm on the left side and 26.0 mm on the right side. 

The following materials were used to fabricate the seven samples examined in this study (framework material + veneering material):

- Gold alloy framework + resin veneering (Au+R);
- Titanium framework + resin veneering (Ti+R);
- CFRC with woven fiber framework + resin veneering (ICFRC+R);
- CFRC with unidirectional fiber framework + composite veneering (UCFRC+C);
- FRC with glass fiber framework + resin veneering (GFRC+R);
- FRC with glass fiber framework + composite veneering (GFRC+C);
- Acrylic resin (fully acrylic prosthesis) (R).

Due to the overlap between composite material science and dental terminology, it must be specified that “resin” was not intended as the polymer matrix that embeds the fibers of the composite material but as the aesthetic material that is supported by the supporting core (framework); similarly “composite” refers to common dental composite material made of a polymeric matrix and inorganic filler, usually applied for the realization of fixed prostheses.

Table 1 reports the dental materials examined in this study (Table 1). 

The guidelines of the respective materials manufacturers were followed for the fabrication of the frameworks and prostheses. Muffle molding or scanning and milling (depending on the materials used) ensured the identical shape of all the prostheses to be tested. 

The gold alloy (Ney-Oro CB, Dentsply Int, York, PA, USA) framework was the first to be fabricated. It was made by the lost-wax casting technique, followed by a passivation technique luting the titanium prosthetic cylinders (Biomet 3i) using an anaerobic composite luting agent, namely BeautiCem SA (SHOFU Dental, Kyoto, Japan) [35,36]. Sandblasting with aluminum oxide (250 µm) and subsequent application of opaquer (SR Ivocron Opaquer Liquid; Ivoclar, Schaan, Liechtenstein) with firing at 100 °C at 6 atm pressure for 10 min was implemented.

The veneer was made of acrylic resin (SR Ivocron, Ivoclar Vivadent, Schaan, Liechtenstein) by clear muffle molding and firing at 120 °C at 6 atm for 15 min, followed by finishing.

For the titanium framework (F.A.B.O.), laser welding of the preformed titanium bars to the titanium cylinders and micro characterization of the framework were performed [27]. The same surface treatment and the same veneering described above for the gold alloy framework were performed. 

Prostheses with a CFRC or a GFRC framework were fabricated following the standard procedures recommended by their manufacturers for dental labs. This strategy was set up instead of procedures for highly specialized engineering-supported labs able to provide state-of-the-art performance from the viewpoint of the Composite Theory but, indeed, far from the experience of the dental community.

To fabricate the CFRC framework made of multidirectional carbon fibers in the fashion of sheets, layers of woven carbon fibers, not pre-impregnated laminae (Dream frame, DEI Italia s.r.l., Mercallo, Varese, Italy), were cut out in a horseshoe shape and soaked with Dream Frame Bio Resin plant-derived epoxy resin (DEI Italia s.r.l. Mercallo, Varese, Italy). Fifteen carbon fiber sheets were superimposed and arranged parallel to the occlusal plane to form the framework in a muffle furnace. The countermold was applied to the model, passing the cylinders between the sheet meshes. Mold firing was performed under vacuum and under pressure at 6 atm at 120 °C for 90 min.

Then, a primer (visio.link PMMA & Composite Primer, Bredent, Bolzano, Italy) was applied, followed by opaquing with SR Ivocron Opaquer Liquid and firing at 100 °C at 6 atm pressure for 10 min.

The veneer was made of resin (SR Ivocron, Ivoclar Vivadent AG, Schaan, Liechtenstein) by clear muffle molding and firing at 120 °C at 6 atm for 15 min, followed by finishing.

Unidirectional carbon fibers (Bio Carbon Bridge Wires, Micro.Medica s.r.l. Palestro, Italy) were uniformly impregnated with Bio Carbon Bridge resin (Micro.Medica s.r.l. Palestro, Italy) and arranged along the entire length of the framework. The countermold was applied to the model, passing the cylinders between the fibers. Mold firing was performed under vacuum and under pressure at 6 atm at 120 °C for 90 min. This was followed by the application of primer (visio.link PMMA & Composite Primer) and opaquer (SR Ivocron Opaquer Liquid), followed by firing at 100 °C at 6 atm pressure for 10 min. 

The veneer was made of composite (SR Nexco Paste, Ivoclar Vivadent AG, Schaan, Liechtenstein). A layer of primer was placed first, followed by light curing in a transparent muffle furnace with a Xenon Lamp Cure System (TOESCO) for 20 min and finishing.

For the glass fiber-reinforced composite (GFRC) framework, preformed Trilor arches (Bioloren S.r.l., Saronno, Va, Italy) constituted of woven glass fibers in an epoxidic resin were milled by CAD/CAM technique. As reported by the manufacturer, Trilor arches present the following mechanical characteristics: density, 1.8 g/cm³; flexural strength, 540 MPa; tensile strength, 380 MPa. Two milled frameworks were realized and luted to the titanium cylinders (Biomet 3i). 

Being whitish in color, no opaque layer is necessary, and only priming is required to promote adhesion of the veneer. 

One GFRC framework was veneered with composite resin (SR Nexco Paste, Ivoclar Vivadent AG Schaan, Liechtenstein) light-cured by transparent muffle molding with a Xenon Lamp Cure System TOESCO for 20 min and finished.

The other was veneered with acrylic resin (SR Ivocron, Ivoclar Vivadent AG Schaan, Liechtenstein), clear muffle-molded, and fired at 120 °C at 6 atm for 15 min, then finished.

All the mechanical tests were performed at the Department of Civil, Chemical, and Environmental Engineering (DICCA) of the University of Genoa.

Fiber composites are not isotropic materials. As a consequence, the mechanical properties of these materials are extremely variable when measured in different directions. This characteristic can be observed either on the microscale, that is, at the fiber level, or on the macroscale, that is, the final product. Thus, the final characteristics of the composite will be influenced by the total fiber percentage, their orientation, and the geometrical arrangement of the layers adopted to create the samples. Due to the experimental nature of these preliminary tests and the specific shapes of implant-supported prostheses, no specific testing protocols or regulatory indications were applied at this stage of the investigation herein described in order to test prostheses realized as in ordinary clinical practice.

All the samples were measured with a caliper to verify that their dimensions could be considered identical and weighed with Exacta Optech precision scales (1000 g full scale, 0.001 g resolution) before compression testing began. The passive fit of each prosthesis was checked using the Sheffield test.

The prostheses were subjected to compression tests using a Zwick/Roell Z 0.5 single-column electromechanical machine with a maximum load of 500 N and 0.1% accuracy.

Before starting the tests, the prostheses were mechanically screwed onto the plaster model using GoldTite retention screws (Biomet 3i) at a torque of 10 N, as recommended by the manufacturer, with a torque instrument (Contra-Angle Torque Driver, Biomet 3i).

Each prosthesis was loaded in 3 different areas at successive times:

- At the first right premolar (equidistant between the retention screw at the lateral incisor level and the one at the first molar level);
- At the first left premolar (equidistant between the retention screw at the lateral incisor level and the one at the first molar level);
- Between the two central incisors (at the interincisal line).

**Figure 2:**
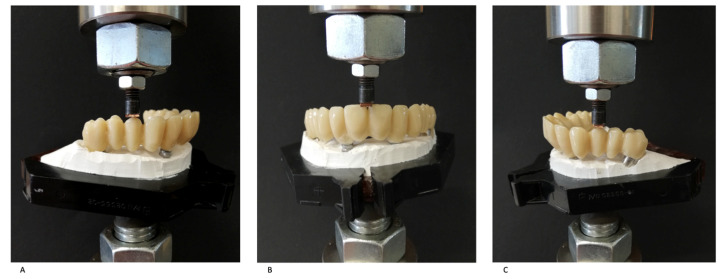
Compression tests at the level of the right (**A**) and left first premolars (**C**) and at the level of the central incisors (**B**).

The purpose of the study was to evaluate the deflection of the prostheses as measured by using a flexural extensometer applied to the lower surface of the sample, following the application of a vertical load (Figure 2). The overall experimental set-up was created in order to test the specific samples herein described (as per clinical-use prostheses).

A 6 mm-diameter cylindrical punch was screwed to the upper load cell to transmit the vertical load (perpendicular to the occlusal plane) by descending onto the prosthesis.

At the sites described above, the three following loading conditions were sequentially applied (Figure 2):

- Increasing load up to 100 N at a speed of 8 mm/min;
- Increasing load up to 200 N at a speed of 16 mm/min;
- Increasing load up to 300 N (for premolar sites) or up to 260 N (for the interincisive site) at 16 mm/min.

The values of 100 N and 200 N were chosen to simulate a typical masticatory load generated during the routine mastication of softer and stiffer food. The forces of 300 N and 260 N correspond to an estimate of the bite force of the posterior region (premolar) and of the anterior segment (intercanine), respectively [37]. 

To ensure uniform load distribution on the surface where the load was applied by the cylindrical punch, due to the irregularity of the aesthetic surface, a thin copper foil with a surface area of 4 mm² and thickness of 1 mm was interposed between the punch and the prosthesis while applying an increasing load up to 100 N and 200 N. On the other hand, when a maximum load of 300 N was applied, a lead foil of the same size was used to avoid damaging the aesthetic coating.

During the application of increasing loading, the deflection of the intaglio surfaces of the prostheses was measured.

The tests were repeated 3 times on all the sites examined under all the loading conditions, and before carrying out the final tests that are reported in this study, several load cycles were established at the value of the final load to allow the copper or lead metal plate to deform, plastically adapting to the profile of the aesthetic part.

## 3. Results

Table 2 reports the weight of the samples and the deflection values of the prostheses (mm) during different load applications on the first left premolar, right premolar, and interincisal line. 

No chipping or fractures of the prostheses occurred during the test.

**Figure 3:**
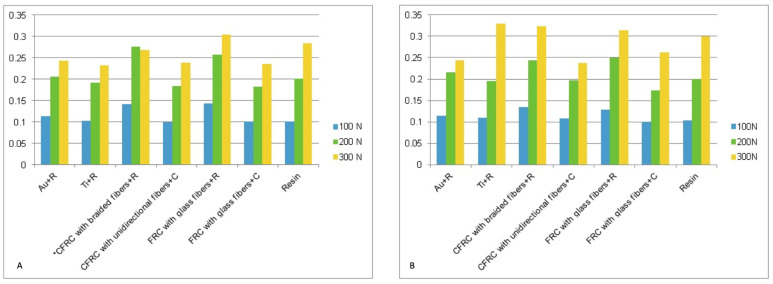
Deformation values in mm for the right (**A**) and left first premolars (**B**).

**Figure 4:**
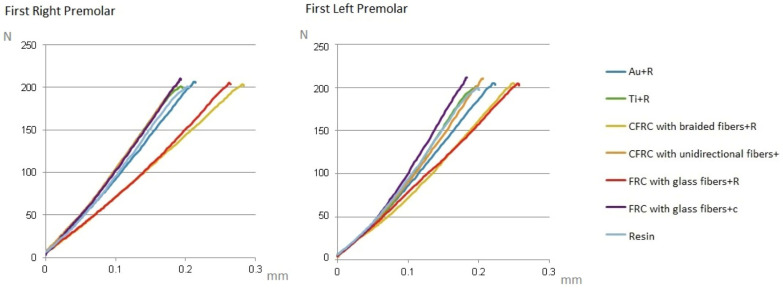
Load/deformation graphs for right and left first premolars.

Compression tests at the level of the right and left first premolars yielded comparable data regarding the mechanical behavior of the tested prosthesis. Table 2 and Figure 3 report the deflection values (mm) of the prostheses collected in the three successive tests (with maximum load at 100, 200, and 300 N) at the level of the right first premolar and at the level of the left first premolar. The load/deflection graphs were obtained on the basis of the collected data (Figure 4).

**Figure 5:**
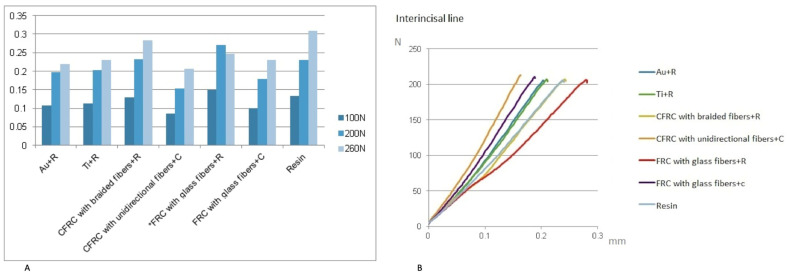
Deformation values in mm (**A**) and load/deformation graphs for the interincisal line (**B**).

Table 2 and Figure 5 report the deflection values of the prostheses (mm) during load application at the interincisal line.

## 4. Discussion

Fiber-reinforced composites (FRCs) differently fashioned in long as well as short fibers and in woven or not-woven configurations have been proposed as an alternative to traditional metal alloys for the realization of frameworks in full-arch implant-supported prostheses. The overall performances of these composite materials are definitely different, but both are applied in dentistry, which pushed us to evaluate their performances.

Gold alloy is the metal that is traditionally used for dentures and bridges, and its properties are proven by long years of use. It is extremely compatible for this type of application, and its mechanical behavior is taken as a reference for evaluating the behavior of other alternative materials [38,39]. 

The traditional gold alloy+resin prosthesis was considered the gold standard in full-arch implant-supported prosthesis, since the metal framework provides strength and rigidly splint implants, while the resinous veneering materials provide shock absorption under occlusal loads for optimal load control [21,27]. 

In the present investigation, the gold alloy+resin prosthesis showed a gradual increase in deflection as the load increased up to 200 N, while at the maximum load of 300 N, it showed a progressive reduction in the deflection gradient as a consequence of an increase in the stiffness. No plastic behavior was observed.

Disadvantages of this material include its high cost and relatively time-consuming and complex fabrication technique (need for casting and passivation technique by luting the titanium cylinders). The latter feature, in particular, is disadvantageous when immediate loading of implants is desired and it is necessary to realize the prosthesis in a short span of time. For these reasons, CAD-CAM techniques are preferred nowadays.

In addition, the prosthesis with the gold alloy framework showed the highest weight among those analyzed, and this represents a further disadvantage in terms of patient comfort. 

Frameworks made of CFRC have been presented as a cheaper alternative to metal alloy frameworks [31,40,41]. In addition to being less expensive, they are easier to manufacture (they do not require casting and special passivation techniques), are lighter, allow for better adhesion of the veneering material (reducing chipping), and do not require special machinery and tools for their fabrication using analogic techniques. As an alternative, disks are available to be milled by applying CAD-CAM technology. 

In a clinical investigation, full-arch prostheses on immediately loaded implants with CFRC frameworks demonstrated less implant failure and significantly less peri-implant bone resorption than full-arch prostheses with metal alloy frameworks [42].

On the other hand, the mechanical properties of this material are operator-dependent and vary considerably depending on the fabrication protocol, material nature, laminate pattern set-up, optimum wetting performances between the fiber and polymer matrix, lab pollution, trapped air bubbles, and the skill of the technician in implementing it; therefore, it is desirable to determine a strict fabrication protocol accompanied by adequate and specific training of the dental technician [36].

While the elastic modulus of isotropic materials from which the framework is made can influence the stress levels transmitted to the implant system and peri-implant bone, in the case of multilayer composites, that can be either unidirectional, multidirectional, or even woven materials, the overall distribution pattern is, indeed, the most important parameter to determine the mechanical behavior under load. This is clinically relevant, since in multiunit prostheses, a deflection of the prosthesis might lead to noxious loads at the abutments and overloading of the implants supporting the prosthesis.

In the present investigation, the multidirection fiber CFRC+resin prosthesis showed stiff behavior but with a greater deflection gradient up to the 200 N load, while at the 300 N load (on dental element 24), an increase in stiffness was observed, presumably due to possible "settling" of the braided fibers but with still greater deformations than the ones observed in the gold alloy prosthesis.

These results are compatible with previous studies; for example, the study by Menini et al. [43] tested prostheses with gold alloy frameworks and prostheses with CFRC frameworks with woven fibers by applying a vertical load identical to that reproduced in this research. The final values demonstrated that the two prostheses exhibited similar behavior and that the least deformation occurred with the gold alloy framework. However, while the prosthesis with a CFRC framework exhibited elastic behavior, recovering its original shape upon removal of the load, the gold+resin prosthesis showed a slight plastic deformation at the end of the load test (maximum load applied: 300 N). In contrast, this phenomenon was not found in the series of tests performed in the present study, where both specimens showed elastic behavior with full recovery of the original sample shape once the load was removed. All these results are, indeed, due to the specific number and orientation set of the multidirectional layers that were adopted in the present study. Any change in the total number of laminae and orientation parameter may result in significantly different conclusions.

The prosthesis with unidirectional fiber CFRC+composite showed lower deformation than the metal alloy and woven CFRC up to a load of 200 N, with a further decrease at 300 N. 

This result confirms the observation described in the study by Pesce et al. [30] that supports the mechanical advantage of the unidirectional fiber CFRC due to the possibility of realizing frameworks with longer whole fibers. In fact, common protocols for making an artifact of a complex shape, such as a full-arch framework, involve cutting out fabrics of pre-interwoven fibers that will then be overlaid. As a result, the fibers in the framework will be shorter. 

Instead, unidirectional fibers can be directly arranged to fit the shape of the framework, which is then traversed along its entire length by whole fibers. 

The unidirectional-fiber CFRC prosthesis weighs slightly more than the multidirectional-fiber CFRC prosthesis, and together, they are the lightest prostheses after the fully acrylic prosthesis. 

In contrast, the titanium prosthesis is slightly heavier than the unidirectional-fiber CFRC prosthesis.

Resin-only and titanium+resin prostheses presented intermediate behavior between unidirectional-fiber CFRC and gold alloy; they exhibited less deflection than the latter up to the 200 N load. When loading at 300 N, an increase in stiffness was observed for titanium (on dental element 14), while, in contrast to the other materials, this phenomenon did not occur in the resin, which presented an increase in strain gradient. 

At low load (100 N), some materials, such as resin, exhibited less ductility than others, such as metal alloy, while at higher load values (200-300 N) the situation was reversed. This can be attributed to the nonlinearity of elastic deformation that occurs, especially for polymeric materials (such as resin) in some specific loading procedures highly dependent upon temperature and testing speed; as the load increases, they progressively reduce the resistance to deformation while remaining in the elastic range.

In addition, the presence of acrylic resin coating instead of composite veneering material may have slightly influenced the deformation values of the prosthesis with a titanium framework.

The FRC prosthesis with glass fibers+resin veneering showed a behavior close to that of woven-fiber CFRC up to a load of 200 N. At a load of 300 N, there was an increase in stiffness. This is due to the less anisotropic behavior of woven CFRC.

In contrast, the FRC prosthesis with glass fiber+composite recorded higher stiffness up to a load of 200 N, with a curve load vs. deflection that remained close to that of unidirectional-fiber CFRCs and titanium. At 300 N (at site 14), there was an increase in stiffness. 

The results in the incisal region were slightly different because the framework was thinner in this area, and there was a different ratio of framework to veneering material, which was more represented than in the premolar region. 

The prostheses with the least deflection at the 200 N load were those made of CFRC with unidirectional fibers+composite and FRC with glass fibers+composite, followed by gold alloy+resin and titanium+resin.

The greatest deformations occurred for resin alone, CFRC with woven fibers+resin, and FRC with glass fibers+resin.

At a load of 300 N, there was an increase in stiffness and, thus, less deformation only in the gold+resin alloy and titanium+resin.

The results of the present research confirm the usefulness of using a rigid substructure in full-arch prostheses. The resin-only prosthesis, in fact, showed high deformation values that might be detrimental, especially in immediate loading rehabilitations, since bending moments would be increased at the implants. Sufficient rigidity of a fully acrylic prosthesis would require greater thicknesses than those simulated in this research. Sufficient prosthetic volume may be present in the case of severe bone atrophy, or it can be achieved by invasive osteoplastic surgery, such as in the all-on-4 technique, where the immediately loaded prosthesis is resin-only [6]. 

Fully acrylic prostheses also have aesthetic disadvantages. In fact, having a greater thickness, they necessarily include a pink resin part to reproduce the soft tissues (Toronto bridge type or “hybrid” prosthesis) [44], moving away from the "Natural bridge" type of prosthesis simulated in the present research that best reproduces the patient’s natural smile. In our study, we assessed prostheses under clinical use conditions, prioritizing analysis of real devices rather than simplified samples, aiming for more accurate and meaningful evaluations.

It must be considered that the flexure of the prosthesis is not only related to the material used, its manufacturing technique, and the thickness of the prosthesis but also to the number of implants, their arrangement, and the length of the span. In the implant-supported rehabilitation herein simulated, with four implants only supporting a full-arch prosthesis, relatively long spans were presented (19.4 mm, 27.6 mm, and 26.0 mm). Testing different configurations, including a greater number of implants and, consequently, shorter spans, a reduced flexure of the prosthesis should be expected. However, together with the number of implants, their arrangement and distribution also significantly affect the mechanical behavior of the prostheses and the resultant forces on the implants. [8] The in vitro study by Ogawa et al. [8] found significantly higher bending moments when the fixed full-arch prosthesis was supported by three implants only instead of four or six and in the case of limited implant distribution. A well-spread polygonal support is considered fundamental in prosthodontics to optimize occlusal load distribution [1].

It is also worth noting that unfavorable stress on the supporting implants might also be due to misfitting of prostheses. All the prostheses tested in the present study were perfectly fit, as recommended for clinical practice. Different outcomes might be expected in the case of misfitting of prostheses, and this might be an interesting topic for future research.

Since precise coupling is a fundamental requirement for long-term success of implant-supported rehabilitations, several techniques have been proposed to achieve passive fitting of full-arch prostheses in the case of immediate loading protocols. Among them, in order to overcome possible errors related to impression techniques and laboratory procedures, splinting the implants by intraoral laser welding has been proposed [45,46,47], although this is not the favorite option according to the present authors and this is why we did not test it.

It must be remembered that many factors must be taken into consideration when choosing the prosthetic material in a specific clinical case. Besides the mechanical aspects investigated in the present research, esthetic needs, the extent of occlusal load (e.g., possible parafunctions), type of antagonist, prosthetic space, prosthesis design, biocompatibility (including influence on biofilm formation), cost, time required for processing, and durability are among the aspects to be considered.

Some limitations of the present investigation must be acknowledged, the first of which is the limited sample size, since only one sample for each material was tested.

Moreover, the samples were all identical in size and shape, although they differed not only in the materials of which they were made but also in the techniques used to fabricating them. Actually, differences in the outcomes may be attributable to the manufacturing process rather than the material. In fact, the prostheses were fabricated for clinical use, and for each material, the techniques recommended by the manufacturers and commonly applied by dental technicians were followed. In addition, the prostheses were screwed to a plaster model during tests, and the peri-implant bone and oral environment were not simulated. 

Given such limitations, this can be considered an exploratory study based on a single sample. Therefore, the outcomes must be considered with caution, and a direct translation to clinical practice guidelines is not possible. On the other side, one of the merits of the present study is the development of an in vitro test protocol for full-arch implant-supported prostheses, where prostheses realized for clinical use were tested and not simplified samples that are far from clinical practice. With the present study set-up, we were able to conveniently investigate the performance of anisotropic materials with respect to the direction of the applied loads.

Further studies might focus on further improvement of the study set-up for a better simulation of the oral environment and evaluation of further significant clinical variables such as biofilm formation and misfitting. In fact, the possible effects of different materials on biofilm formation were not investigated in the present study, although plaque accumulation and biofilm composition might significantly affect the long-term success of rehabilitation.

## 5. Conclusions

In conclusion, the present mechanical investigation demonstrated that fiber-reinforced composites can be a viable alternative to metal alloys for the fabrication of frameworks for full-arch implant-supported prostheses, presenting additional advantages such as lower cost, ease of repair, and lower weight. 

Frameworks made of unidirectional-fiber CFRC demonstrated lower deflection under load compared to woven CFRC.

As for glass fiber-reinforced composites, further studies are needed in order to investigate their mechanical properties in implant-supported prostheses in relation to the prosthesis thickness.

Resin alone, at the thicknesses simulated in the present research, does not provide a sufficiently rigid structure to ensure sufficient strength and rigidity for full-arch implant-supported rehabilitations.

## Figures and Tables

**Table 1:** Dental materials examined in this study.

Material	Trade Name	Manufacturer
Titanium	-	F.A.B.O.
Gold alloy	Ney-Oro CB	Dentsply Int
CFRC with multidirectional fibers	Dream Frame	DEI Italia s.r.l.
CFRC with unidirectional fibers	Bio Carbon Bridge	Micro.Medica s.r.l.
FRC with glass fibers	TRILOR ARCH	Bioloren S.r.l
Composite	SR Nexco Paste	Ivoclar Vivadent AG
Resin	SR Ivocron	Ivoclar Vivadent AG

**Table 2:** Weight of each prosthesis and deflection values of the prostheses (mm) during different load applications on the first left premolar, first right premolar, and interincisal line.

		First Left Premolar			First Right Premolar			Interincisal Line		
	Weight (g)	100N	200N	300N	100N	200N	300N	100N	200N	260N
Gold alloy+resin	28.4	0.115	0.215	0.243	0.114	0.206	0.244	0.108	0.198	0.219
Titanium+resin	12.1	0.110	0.195	0.329	0.103	0.192	0.232	0.114	0.202	0.230
CFRC with braided fibers+resin	10.1	0.134	0.243	0.323	0.141	0.276	0.268 *	0.129	0.233	0.284
CFRC with unidirectional fibers+composite	11.8	0.109	0.197	0.237	0.099	0.184	0.239	0.085	0.154	0.207
FRC with glass fibers+resin	10.5	0.128	0.250	0.314	0.144	0.257	0.304	0.149	0.270	0.247 **
FRC with glass fibers+composite	14.4	0.101	0.174	0.262	0.101	0.183	0.235	0.100	0.179	0.230
Resin	9.3	0.104	0.200	0.300	0.101	0.201	0.284	0.133	0.231	0.310

* The value at 300 N is not significant because it is related to machine settling. ** The value at 260 N is not significant because it is related to machine settling.

## Data Availability

Data are available upon request.

## Abbreviations

gold alloy+resin (Au+R), titanium+resin (Ti+R), FRC with multidirectional carbon fibers+resin (ICFRC+AR), FRC with unidirectional carbon fibers+composite (UCFRC+C), FRC with glass fibers+resin (GFRC+AR), FRC with glass fibers+composite (GFRC+C), resin (R, full-acrylic prosthesis).
